# Burst mode pumping: A new mechanism of drinking in mosquitoes

**DOI:** 10.1038/s41598-018-22866-w

**Published:** 2018-03-20

**Authors:** Kenji Kikuchi, Mark A. Stremler, Souvick Chatterjee, Wah-Keat Lee, Osamu Mochizuki, John J. Socha

**Affiliations:** 1 Department of Finemechanics, Tohoku University, Sendai, 980-8579 Japan; 2 Department of Biomedical Engineering and Mechanics, Virginia Tech, Blacksburg, VA 24061 USA; 3 National Synchrotron Light Source II, Brookhaven National Laboratory, Upton, NY 11937 USA; 4 Toyo University, Saitama, 350-8585 Japan

## Abstract

Mosquitoes transport liquid foods into the body using two muscular pumps in the head. In normal drinking, these pumps reciprocate in a stereotyped pattern of oscillation, with a high frequency but small stroke volume. Do mosquitoes modulate their neuromotor programs for pumping to produce different drinking modes? More broadly, what are the mechanical consequences of a two-pump system in insects? To address these questions, we used synchrotron x-ray imaging and fluid mechanical modeling to investigate drinking performance in mosquitoes. X-ray imaging of the pumps during drinking revealed two modes of pumping: continuous reciprocation with multiple small strokes, and a newly discovered ‘burst mode’ involving a single, large-volume stroke. Results from modeling demonstrate that burst mode pumping creates a very large pressure drop and high volume flow rate, but requires a massive increase in power, suggesting that continuous pumping is more economical for drinking. Modeling also demonstrates that, from one mode of pumping to the other, the mechanical role of the individual pumps changes. These results suggest that the advantage of a two-pump system in insects lies in its flexibility, enabling the animal to pump efficiently or powerfully as demanded by environmental considerations.

## Introduction

Mosquitoes are one of many species of fluid-feeding insect. It is well known that only female mosquitoes ingest blood, which is needed to produce eggs^1^. However, all adult moquitoes drink nectar for nourishment. For either fluid, there are potential advantages for the mosquito to drink more or drink faster. When a mosquito inserts its proboscis into a host to drink, there is a limited time window available before the onset of irritation and detection by the host (on the order of 3 minutes for humans^2^). Interruption of feeding or injury to the mosquito can lead to smaller blood meals and fewer mature eggs^3^, providing selection pressure for faster drinking performance. Larger blood meals result in increased egg production, contributing to greater fecundity and fitness^4^. Fast drinking of nectar would result in briefer drinking bouts, potentially lowering risk of predation.

In theory, to feed faster a mosquito could reduce the time to reach its maximum intake rate or increase its average intake rate, both of which concern the volume flow rate^3^. Creating higher volume flow rates would lead to shorter drinking bouts and would therefore benefit the mosquito for avoiding detection or predation. Controlling the volume flow rate, therefore, is a key factor in determining drinking performance in mosquitoes, with direct implications for the insect’s ecology. However, flow rate in a drinking mosquito is constrained by the high resistance to flow through the tiny food canal in the proboscis (canal diameter, ~25 µm) and the operating pattern of the pumps in the head, making pumping an energetically demanding process^5–7^. The small size of the canal may also render the feeding system susceptible to problems of obstruction due to small particles or air bubbles.

**Figure 1:**
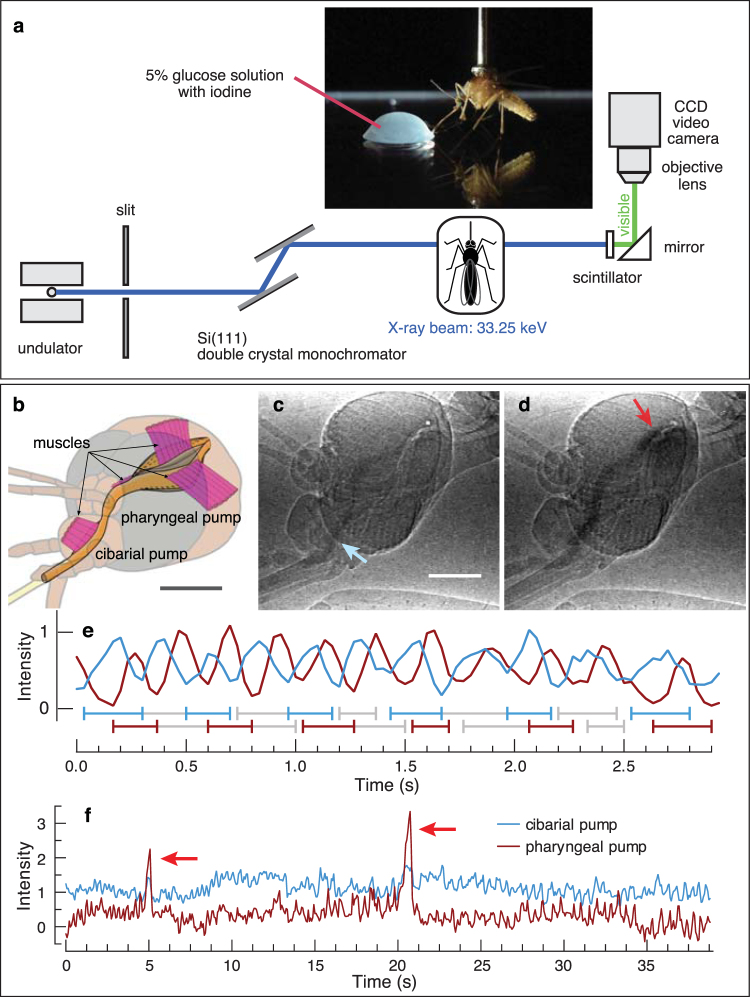
Experimental measurement of pumping using synchrotron x-ray imaging. (**a**) Schematic of the synchrotron x-ray imaging setup at beamline 32-ID-C at the Advanced Photon Source, Argonne National Laboratory. (**b**) Location of the cibarial and pharyngeal pumps, and associated major musculature. (**c**) Still frame from an x-ray video showing a darkened line of sugar water/iodine in the feeding system during continuous pumping. Blue arrow, cibarial pump. Scale bar, 200 µm. (**d**) Full volume expansion of both pumps during a burst event, 0.5 s after the previous image. Red arrow, pharyngeal pump. (**e**) Details of continuous pumping, with fill-ejection timing for each pump shown below the trace (every other cycle shown). Dimensionless intensity values represent the volume of the feeding solution, with raw values normalized by the maximum and minimum values from a sequence. (**f**) A long bout of continuous pumping interspersed with two burst events (red arrows). The intensity values for the cibarial pump have been shifted upward on the graph for clarity.

Mechanically, the pressure drop across the proboscis and the corresponding volume flow rate of drinking are determined by the morphology and dynamics of the upstream components of the digestive system. Nectar or blood enters through the narrow food canal in the proboscis, which connects to two in-line pumps in the head (Fig. 1b). These pumps provide the motive force that drives flow through the food canal. The cibarial pump, which lies upstream, is smaller than the pharyngeal pump, which sends food to the esophagus and the rest of the digestive system. Between the pumps lies the pharynx, which acts as a valve^8,9^. During feeding, the two pumps in the head of the mosquito expand and contract rapidly multiple times per second, with expansion driven by muscles (Fig. 1b) and contraction occurring by elastic recoil^10^. Electrophysiological recordings have shown that pumping begins out of phase and then settles quickly (~5–8 contractions) into a cyclic pattern in which strokes of the pharyngeal pump follow those of the cibarial pump with a phase lag of 10–30%^11^. Although a mosquito could theoretically alter its feeding performance by changing the frequency, timing, or stroke volume of pumping, only one program of pumping has been observed previously^11–13^. Based on principles of fluid mechanics, the pumps must produce a pressure drop across the ends of the food canal to produce an intake flow. Variation in expansion rate and volume of the pumps, combined with variation in their phasing and valving, should result in variable pressure drops, and hence differences in flow rate or blockage-clearing ability. However, the mechanical consquences of variation in operation of the mosquito pumping system, or any two-pump system in insects, remain obscure.

Here, we use experimental measurements of drinking combined with mathematical modeling of the fluid flow to understand the mechanics of drinking in mosquitoes. Specifically, we ask, do mosquitoes modulate pumping parameters to control intake flows during drinking? The visualization of pumping movements within the head have previously been prohibited due to the opacity of the insect’s exoskeleton, but recent work has demonstrated the use of synchrotron x-rays to image fluid flows in the digestive systems of insects^14,15^, including mosquitoes^9,12^. We used synchrotron x-ray imaging and mathematical modeling to observe and analyze, respectively, the drinking strategies of mosquitoes. In addition, we used the model to probe the mechanical significance of a two-pump system in insects.

## Methods

### Overview

We observed internal pumping movements in the heads of female mosquitoes using synchrotron x-ray imaging at the Advanced Photon Source, Argonne National Laboratory. X-ray videos showing the feeding solution moving through each mosquito’s head were created using x-rays tuned just above the iodine K-absorption edge, which rendered the food opaque^14^. We also used synchrotron tomography to determine the 3D morphology of the major features of the head. Lastly, using parameters from the observed morphology and kinematics, we developed a mathematical model of fluid pumping by a mosquito.

### Animals

Two species of mosquitoes from the genus *Aedes* (*A. vexans* and *A. cinereus*) were used for live imaging studies. Mosquitoes were wild-caught locally using traps. In the period prior to experimentation, mosquitoes were kept in small cages with *ad libitum* food and water, and then were starved for up to 48 hours directly before a feeding trial. Only females were used, and data were considered at the genus level (i.e., all data were pooled).

### X-ray visualization of mosquito drinking

X-ray imaging of drinking mosquitoes was performed at the XOR 32-ID-C beamline at the Advanced Photon Source. To prepare for imaging, mosquitoes were anesthetized with nitrogen gas and glued to the head of an insect pin at the dorsal thorax using clear nail polish (Hard as Nails Strengthening Topcoat, Sally Hansen, New York, NY, USA). The pin was attached to a mechanical manipulator to control the position and orientation of the mosquito (Fig. 1a). The mosquito was placed upright on a glass slide and the tip of its proboscis was manually submerged into a drop of feeding solution, stimulating the sugar-sensitive labellar or labral sensilla^11^ to induce pumping. The feeding solution was a 1:1 solution of 10% glucose solution and an iodine contrast agent (Isovue 370, Bracco Diagnostics, USA), yielding a net 5% glucose solution with contrast-enhancing capabilities. The glass slide was located on a motorized stage that permitted the mosquito to be accurately positioned within the x-ray beam. Each mosquito was positioned such that the projection images were taken perpendicular to the flow direction (i.e., in lateral view). Drinking occurred in 58% (36 of 62) of the specimens.

We recorded x-ray videos of drinking using the following setup. We used monochromatic x-rays (33.25 keV), tuned just above the iodine K-absorption edge, to visualize the feeding solution moving through the mosquito’s head^14^. A cerium-doped yttrium aluminum garnet single-crystal scintillator converted the transmitted x-rays into visible light (550 nm), which was imaged onto a CCD video camera (Cohu 2700, 768 (H) × 494 (V) pixels, 8.4 µm (H) × 9.8 µm (V) pixel size, Cohu Inc., USA) via a 5× microscope objective/tube lens combination. The analog output of the camera was recorded onto miniDV tapes at 30 frames per second with an image size of 720 × 480 px. The field of view was 1.3 × 1.0 mm (H × V). A gold 400-mesh transmission electron microscope grid was imaged for spatial calibration.

### Video analysis

Videos were downloaded onto a Macintosh computer using Final Cut Pro software and converted to.mov format. Videos were analyzed using ImageJ software^16^. For each trial, two regions of interest (ROI) were defined based on the approximate dimensions of the maximum expanded volume for each pump (cibarial and pharyngeal pumps; see Supplementary Fig. S1). At each time step (Δt = 1/30 s), the average grayscale intensity within the ROI was determined. Because the absorption of x-rays is a linear function of mass, these intensity values provide a measure of the volume of feeding solution, and therefore constitute the raw data for each pump. Intensity data were smoothed using a 3-point moving average and then normalized using the minimum and maximum intensity values observed for each bout of continuous pumping (Fig. 1e,f). The start, peak, and end of each pump stroke were identified from local minima and maxima in the time-series plots. These values were used to calculate pump cycle timing variables, reported in Table 1. The duration for each pump was calculated using their respective start and end times. The total cycle duration was defined as the time from the start of the cibarial stroke to the end of the pharyngeal stroke. The duration between cycles was defined as the time between the end of the pharyngeal stroke to the beginning of the next cycle’s cibarial stroke.

**Table 1:** Summary of timing characteristics of continuous and burst mode pumping, determined by measurements from x-ray videos of drinking.

	Continuous mode pumping		Burst mode pumping	
	Absolute (ms)	Relative (%)	Absolute (ms)	Relative (%)
***Event timing variables***				
CP Start time	1 ± 2	0.2 ± 0.6	0.4 ± 1.3	0.1 ± 0.2
PP Start time	92 ± 40	27.1 ± 6.6	153 ± 66	22.6 ± 6.4
CP Peak time	135 ± 44	41.4 ± 3.1	449 ± 278	59.9 ± 12.5
PP Peak time	204 ± 64	63.3 ± 3.7	498 ± 244	69.6 ± 5.7
CP End time	242 ± 66	76.8 ± 6.4	662 ± 310	92.1 ± 7.0
PP End time	318 ± 91	99.5 ± 0.9	646 ± 244	93.6 ± 6.3
***Duration variables***				
Total pumping cycle	319 ± 91	100 ± 29	703 ± 288	100 ± 41
CP pump cycle	241 ± 65	76.6 ± 6.4	661 ± 310	92.0 ± 7.0
PP pump cycle	226 ± 52	72.4 ± 6.7	493 ± 192	71.0 ± 7.1
Between pump starts	91 ± 40	27.0 ± 6.3	152 ± 67	22.5 ± 6.5
Between pump peaks	69 ± 24	21.7 ± 4.3	49 ± 61	9.7 ± 8.6
Between pump ends	76 ± 34	22.6 ± 6.8	−16 ± 98	1.5 ± 12.2
Time to next pumping cycle	−44 ± 18	−14 ± 3.9	N/A	N/A
***Summary variables***				
Total pumping cycle frequency (Hz)	4.0 ± 1.4	N/A	N/A	N/A
CP:PP duration ratio	1.09 ± 0.08	N/A	1.35 ± 0.23	N/A

Total cycle duration refers to the total pumping action of the cibarial and pharyngeal pumps, representing one full stroke of each pump. Data are given in absolute durations in milliseconds, and relative durations as percentage of the total cycle duration. The values above were calculated using average values per specimen. Data from continuous pumping events derive from 126 cycles from six specimens (average, 21 cycles/specimen), and the burst mode pumping events are from 31 cycles from 10 specimens (average, 3.1 cycles/specimen).

### Statistical analyses

Comparisons of timing and duration of pumping events across anatomical pumps and pumping modes were conducted using mixed models using R (ver. 3.3.1) and RStudio (ver. 0.99.903) on a Macintosh computer. Pumping mode and anatomical pump were considered as fixed factors, and specimen was considered as a random factor.

### X-ray visualization of mosquito head anatomy using synchrotron microcomputed tomography (SR-µCT)

Tomographic x-ray imaging of mosquito heads was performed at the XOR 2-BM-B beamline at the Advanced Photon Source, Argonne National Laboratory, following Socha *et al*.^17^. Mosquitoes of the species *Aedes vexans* were caught locally, sacrificed immediately using ethyl acetate, and mounted at the ventral thorax using an insect pin and clear nail polish. Two-dimensional projection images created with pink beam x-rays (E = 10–30 keV) were collected at rotation steps of 0.125° and recorded using a high speed camera (pco.dmax, PCO-TECH Inc., Romulus, MI, USA). The scintillator that converted x-rays to visible light was a cerium-doped lutetium aluminum garnet crystal, located at a distance of 60 mm from the sample. Reconstruction from raw 2D projection images to 3D slice images was conducted using TomoPy software^18^ at the Advanced Photon Source. Three-dimensional rendering and image segmentation were conducted using Avizo software (FEI Visualization Sciences Group, Burlington, MA, USA). A total of three mosquitoes were scanned.

#### Determining morphological dimensions and temporal kinematics

**Figure 2:**
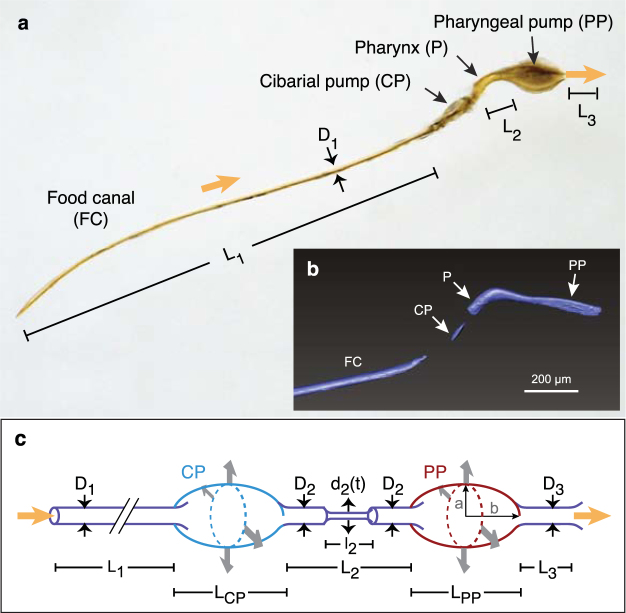
The feeding system in the proboscis and head of the mosquito. (**a**) The food canal, cibarial pump, pharynx, and pharyngeal pump, dissected from a specimen of *Aedes albopictus*. (**b**) Three-dimensional volume rendering of the feeding system in the posterior proboscis and head of *Aedes vexans*, generated from synchrotron x-ray µCT data. Scale bar, 200 µm. (**c**) Schematic of the feeding system, showing the variables used to create the mathematical model of pumping. *D*_1_ and *L*_1_ are the diameter and length of the proboscis; *L*_CP_ and *L*_PP_ are the lengths of the cibarial and pharyngeal pumps, respectively; *D*_2_ and *L*_2_ are the diameter and length of the pharynx; *D*_3_ and *L*_3_ are the diameter and length of the esophagus in the head; *d*_2_ and *l*_2_ are the diameter and length of the constricting section in the pharynx to simulate valving; *a* and *b* are the minor and major axes of the prolate spheroid that represents a pump.

The morphological dimensions and temporal kinematics of the drinking process in a representative mosquito were estimated based on data from the x-ray videos (Supplementary Videos S1–S4), the tomographic x-ray images (segmentation shown in Supplementary Fig. S2, Supplementary Video S5), and knowledge of mosquito anatomy from dissection (e.g., Fig. 2a) and the literature^10^.

Average dimensions were determined for the relevant anatomical features labeled in Fig. 2 and are summarized in Table 2. The length (*L*_1_) and diameter (*D*_1_) of the feeding canal (FC) were measured via microscope to be approximately *L*_1_ = 1.56 mm (n = 8) and *D*_1_ = 25 µm, respectively. The length of the pharynx (P) was observed from the x-ray videos to be approximately 8 times smaller than that of the proboscis, or *L*_2_ = 200 µm (n = 4). Based on the video evidence, the diameter of the pharynx appeared to be approximately the same as that of the feeding canal; we took *D*_2_ = 25 µm. The exact dimensions of the esophagus during feeding were difficult to determine from the x-ray video; the diameter appeared to be larger than that of the pharynx and but not significantly greater than the width of the pharyngeal pump in continuous-mode operation. We estimated as best possible a length *L*_3_ = 100 µm and a diameter *D*_3_ = 50 µm for the esophagus. These estimates resulted in the esophagus providing relatively low resistance to the flow of food to the gut in the mathematical model, in which case the exact dimensions have little effect on the model results.

**Table 2:** Morphological dimensions of the proboscis, pharynx, and esophagus used in the modeling analysis.

	Length (μm)	Diameter (μm)
Proboscis	*L*_1_ = 1560 (n = 8)	*D*_1_ = 25
Pharynx	*L*_2_ = 200 (n = 4)	*D*_2_ = 25
Pharyngeal valve (closed)	*l*_2_ = 20	*d*_2_ = 5
Esophagus	*L*_3_ = 100	*D*_3_ = 50

Two valves in the system are thought to provide some level of flow control: one in the pharynx (pharyngeal valve)^8^ and one at the entrance to the esophagus (esophogeal valve)^19^. Although these valves are known from morphological^8,19^ and functional investigation^9^, the literature does not contain detailed descriptions of the dynamics of either valve, so we assumed a form and function based on prevention of backflow in the system. Previous anatomical images^8^ show that the pharyngeal valve does not extend the length of the pharynx, but accurate dimensions and timing of operation are not known. For the purposes of this investigation, we estimated that the pharyngeal valve can be represented by having a length *l*_2_ = 20 µm of the pharynx constrict to a diameter *d*_2_ = 5 µm in response to backflow through the pharynx. For the esophagus, we simply assumed that no backflow occurred, so valve dimensions were not needed. Note that the assumed form and function of the pharyngeal and esophageal valves do affect the details of the modeling results. Similarity between our modeled proboscis flow and the velocity measurements in a previous study^20^ support the validity of our valve assumptions. Furthermore, it is reasonable to assume that the valves operate in the same way in both of the drinking modes, so that the comparative behavior of these modes, as revealed by the modeling, remains consistent even with moderate changes in the valve details.

The flow of liquid food through the feeding canal and into the gut is driven by motion of the cibarial pump (CP) and the pharyngeal pump (PP). Time-dependent sizes of the pumps were estimated by observing the darkened regions caused by the presence of feeding solution containing iodine-contrast agent, as illustrated in Fig. 1c,d. Pump dimensions were measured directly from frames of the x-ray videos. The lengths of these pump segments, *L*_CP_ and *L*_PP_ (equivalent to twice *b* in Fig. 2c), appeared to remain essentially constant throughout feeding. We measured these lengths in frames of the x-ray videos that show near-maximum expansion of each pump. As reported in Table 3, we found *L*_CP_ = 232 µm (n = 4) and *L*_PP_ = 326 µm (n = 4).

**Table 3:** Morphological dimensions of the head pumps used in the analytical model.

	Cibarial pump	Pharyngeal pump
Length (μm)	${L}_{{\rm{CP}}}=$ 232 (n = 4)	${L}_{{\rm{PP}}}=$ 326 (n = 4)
Height, resting state (μm)	${({H}_{{\rm{CP}}})}_{{\rm{\min }}}=$ 38 (n = 4)	${({H}_{{\rm{PP}}})}_{{\rm{\min }}}=$ 44 (n = 1)
Height, continuous mode expanded state (μm)	${({H}_{{\rm{CP}},{\rm{C}}})}_{{\rm{\max }}}=$ 50	${({H}_{{\rm{PP}},{\rm{C}}})}_{{\rm{\max }}}=$ 50
Height, burst mode expanded state (μm)	${({H}_{{\rm{CP}},{\rm{B}}})}_{{\rm{\max }}}=$ 63 (n = 4)	${({H}_{{\rm{PP}},{\rm{B}}})}_{{\rm{\max }}}=$ 256 (n = 4)

The height of each pump, *H*_CP_ and *H*_PP_ (equivalent to twice *a* in Fig. 2c), was observed to vary in time according to the mode of drinking. The minimum and maximum values of these dimensions, which we refer to as the pump heights, are key factors in characterizing the system behavior for each drinking mode. When determined using the x-ray data, a pump height value for a single specimen at a given instant in time was taken to be an average of three measurements from different locations along the length of the pump.

The minimum values of *H*_CP_ and *H*_PP_ for both modes of pumping were determined using a combination of x-ray and tomography data. For the cibarial pump, we determined the minimum value from frames of the x-ray video that showed the smallest amount of feeding solution, giving (*H*_CP_)_min_ = 38 μm (n = 4). The minimum value of the pharyngeal pump height was difficult to identify from the x-ray videos. Therefore, we used tomography data from a single mosquito to estimate (*H*_PP_)_min_ = 44 μm (n = 1). This estimate assumes that the pumps of a sacrificed specimen are in the minimally-expanded state.

The maximum values of *H*_CP_ and *H*_PP_ were found using x-ray data and estimation. In the burst mode, these heights were determined by direct measurement of x-ray video images, giving (*H*_CP,B_)_max_ = 63 µm (n = 4) and (*H*_PP,B_)_max_ = 256 μm (n = 4) for the cibarial and pharyngeal pumps, respectively, as reported in Table 3. During the continuous mode of drinking, distinct boundaries could not be clearly identified in the x-ray images for either pump. The video data show that cibarial pump expansion is less in the continuous mode than in the burst mode, and we estimated from observation of the video sequences that (*H*_CP,C_)_max_ is roughly 80% of (*H*_CP,B_)_max_, i.e. that (*H*_CP,C_)_max_ = 50 µm. Considering that the pharyngeal pump behavior appears similar to that of the cibarial pump during this mode, we also assumed (*H*_PP,C_)_max_ = 50 μm.

### Mathematical modeling of fluid motion

Estimates of fluid flow rates and internal pressures in and through the mosquito drinking system were obtained by fitting a reduced-order mathematical model to the experimental data. The model also allowed for predictions of the mechanical power transferred to the fluid by each pumping mode and for performance comparisons with comparable single-pump systems.

The feeding solution, a net 5% glucose solution as described previously, was assumed to be incompressible and behave as a Newtonian fluid^21^, with a linear relationship between applied stress and the resulting strain rate. Based on composition, we estimated the solution density to be ρ = 1.1 g/cm^3^. At 22.5 °C, the temperature at which the mosquitoes were fed, the dynamic viscosity of the sugar/iodine solution was measured to be μ = 3.0 ± 0.05 cP in a cone-plate viscometer (DV-II + Pro, Brookfield, Engineering Laboratories, Middleboro, MA, USA). Previous measurements in a mosquito proboscis^20^ suggest that the average fluid velocity, *U*, in the feeding canal does not exceed 1 cm/s. Thus, the Reynolds number in the feeding canal, Re = ρ*UD*1/μ, where *D*_1_ = 25 μm (Table 2), was estimated to be Re ≈ 0.1, well within the range needed to satisfy a laminar flow assumption.

The importance of considering the pulsatory nature of the flow through the feeding canal and the other small tubes in the system was quantified by the Womersley number^22^, *α* = (ρ*D*_*i*_^2^/μ*T*)^1/2^, where *T* is the time period of the pulsatile flow and *D*_*i*_ is the relevant tube diameter. Using time periods and tube diameters from Tables 1 and 2, we estimated *α* < 0.1 throughout the feeding system, showing that pulsatile effects can be neglected^22^ when considering the flow through the feeding canal, pharynx, and esophagus. A similar rationale has been used for modeling single-pump drinking systems^23^.

The modeling analysis considered flow of the feeding solution through a simplified representation of the mosquito feeding system (Fig. 2c). For the cases being modeled, the mosquito is in the midst of a bout of drinking, so the fluid solution was assumed to entirely fill the feeding system, neglecting the influence of any bubbles or capillary effects due to free surfaces. The feeding canal, pharynx, and esophagus were treated as straight tubes with circular cross section. The pumps were assumed to be prolate spheroids with minor axes (*a*_1_, *a*_2_) that vary as sinusoidal functions of time. The dimensions of the system were based on the anatomy of the mosquito (Fig. 2a,b; Tables 2 and 3), and the pump wall motions were based on the kinematics observed during the drinking experiments (Table 1).

The fluid motion was modeled using a control volume analysis, a standard approach to determining the averaged dynamics in a system^21^. Fluid mass must be conserved as it passes through each portion of the system. Because the fluid density was assumed to be constant, mass conservation in the tubes representing the feeding canal, pharynx, and esophagus requires that the time-dependent volumetric flow rates, *Q*_*i*_(t), remain constant along their length, where the notation *i* = 1 represents the feeding canal, *i* = 2 the pharynx, and *i* = 3 the esophagus. In the pumps, mass conservation is equivalent to volume conservation, giving

1 $${\dot{V}}_{\beta }(t)={Q}_{{\rm{in}},\beta }(t)-{Q}_{{\rm{out}},\beta }(t)$$

where ${\dot{V}}_{\beta }$ is the time rate of change of volume in pump *β*, with *β* = CP indicating the cibarial pump and *β* = PP the pharyngeal pump. The relevant flow rates in and out of the pumps are *Q*_in,CP_ = *Q*_1_, *Q*_out,CP_ = *Q*_in,PP_ = *Q*_2_, and *Q*_out,PP_ = *Q*_3_.

Linear momentum conservation was used to determine the relationships between pressures and flow rates. For the pumps, we assumed that the pressure, *p*_*β*_(*t*), is uniform throughout the pump volume. In essence, this modeling step assumes that the fluid in the pump responds instantly to variations in the pump walls, so that at any instant in time the fluid in each pump is essentially static. This quasi-static assumption greatly simplifies the analysis, but it neglects energy losses in the pumps and is likely the largest source of error in this reduced-order model. The time-dependent pump pressures drive fluid through the tubes representing the feeding canal, pharynx, and esophagus, for which we assumed the flow rate, *Q*_*i*_(t), to be linearly proportional to the pressure drop over the tube length, Δ*p*_*i*_(*t*). The relevant pressure drops are Δ*p*_1_ = *p*_food_ − *p*_CP_, Δ*p*_2_ = *p*_CP_ − *p*_PP_, and Δ*p*_3_ = *p*_PP_ − *p*_gut_, where *p*_food_ is the pressure of the food at the entrance to the proboscis and *p*_gut_ is the pressure of the food in the gut at the exit of the esophagus. We write this Hagen-Poiseuille flow model^21,24^ as

2 $${\rm{\Delta }}{p}_{i}(t)={K}_{i}(t)\,{Q}_{i}(t),$$

which is valid for steady, laminar flow in straight, circular tubes. We allowed for the flow impedance values, *K*_*i*_(*t*), to be time dependent to account for the effects of internal valving on flow control. Based on the estimate of Re ≈ 0.1 in the feeding tube, we assumed that the flow remains laminar throughout the entire feeding system. The Womersley number estimate of *α* < 0.1, which shows that the time scale for changes in the pressures and flow rates is much larger than the time scale for viscous dissipation across each tube, justifies using a quasi-steady model of flow through each tube.

The control volume analysis produces a mathematical model consisting of five equations for the five variables *p*_*β*_(*t*), *β* = CP, PP and *Q*_*i*_(*t*), *i* = 1, 2, 3. Solving this system gives the flow rates through the feeding canal, pharynx, and esophagus as, respectively,

3a $$(\begin{array}{c}{Q}_{1}(t)\\ {Q}_{2}(t)\\ {Q}_{3}(t)\end{array})=-\frac{{\rm{\Delta }}{p}_{g}}{\sum {K}_{i}(t)}\,{\bf{1}}+\frac{{\dot{V}}_{{\rm{CP}}}(t)}{\sum {K}_{i}(t)}(\begin{array}{c}{K}_{2}(t)+{K}_{3}(t)\\ -{K}_{1}(t)\\ -{K}_{1}(t)\end{array})+\frac{{\dot{V}}_{{\rm{PP}}}(t)}{\sum {K}_{i}(t)}(\begin{array}{c}{K}_{3}(t)\\ {K}_{3}(t)\\ -{K}_{1}(t)-{K}_{2}(t)\end{array}).$$

where **1** is the identity vector, $\sum {K}_{i}(t)={K}_{1}(t)+{K}_{2}(t)+{K}_{3}(t)$, and Δ*p*_*g*_ = *p*_gut_ − *p*_food_ represents a backpressure in the system from the gut. The solution also gives the pressures in the cibarial and pharyngeal pumps as

3b $$\begin{array}{rcl}(\begin{array}{c}{p}_{{\rm{CP}}}(t)\\ {p}_{{\rm{PP}}}(t)\end{array}) & = & {p}_{{\rm{food}}}\,{\bf{1}}+\frac{{\rm{\Delta }}{p}_{g}}{\sum {K}_{i}(t)}(\begin{array}{c}{K}_{1}(t)\\ {K}_{1}(t)+{K}_{2}(t)\end{array})-\frac{{K}_{1}(t)\,{\dot{V}}_{{\rm{CP}}}(t)}{\sum {K}_{i}(t)}(\begin{array}{c}{K}_{2}(t)+{K}_{3}(t)\\ {K}_{3}(t)\end{array})\\  &  & -\frac{{K}_{3}(t)\,{\dot{V}}_{{\rm{PP}}}(t)}{\sum {K}_{i}(t)}(\begin{array}{c}{K}_{1}(t)\\ {K}_{1}(t)+{K}_{2}(t)\end{array}),\end{array}$$

The maximum pressure drop over the length of the proboscis is given by

4 $${\rm{\Delta }}{p}_{\gamma }=\,{\rm{\max }}\{|{p}_{{\rm{CP}}}(t)-{p}_{{\rm{food}}}|\},$$

where *γ* = B, C will be used to indicate the drinking mode. Estimates of the fluid dynamics in the feeding system can thus be determined by specifying the pressures in the gut and food, the time-dependent variation in the pump volumes, ${\dot{V}}_{\beta }(t)$, and the tube impedance values, *K*_*i*_(*t*).

For the analysis presented in this manuscript, we assumed that the fluid pressures at the inlet and exit of the model system (in the food and gut, respectively) and in the hemolymph surrounding the system were equal to the (constant) ambient atmospheric pressure, *p*_atm_. The food source was open to the atmosphere, and we neglected the small pressure increase in the food droplet due to surface tension (estimated to be less than 3 Pa in a droplet with a diameter of 7 mm), giving the inlet condition *p*_food_ ≈ *p*_atm_. Measurements in other insect species show baseline hemolymph pressures that are near-atmospheric^25,26^. We were not able to measure the hemolymph pressure in the head, so we assumed that hemolymph pressures were atmospheric (*p*_hemo_ = *p*_atm_) for the mosquito. Prior to abdominal expansion, the gut pressure is thus also likely to be atmospheric. While some backpressure from the gut may develop as the mosquito feeds and the abdomen swells, such pressures have not been measured. Therefore, we assumed that food exits the model esophagus into a gut for which *p*_gut_ ≈ *p*_atm_, so that the gut backpressure was taken to be Δ*p*_*g*_ = 0.

The exact geometry of each pump during operation is not well established in the literature. The best indication of its static shape can be found in Figure S2 and a previous study^9^, but its time-dependent shape is unknown. Given its morphology, we assumed that the shape of each pump could be approximated as a prolate spheroid with volume

5 $${V}_{\beta }(t)=\frac{4\pi }{3}\,{a}_{\beta }^{2}(t)\,{b}_{\beta },$$

where *a*_*β*_(*t*) and *b*_*β*_ are the semi-minor and semi-major axes of the spheroid, respectively. We assumed *b*_*β*_ = ½ *L*_*β*_ was a constant value throughout the drinking cycle for both drinking modes and both pumps, where *L*_*β*_ is the experimentally determined length of the corresponding pump (Table 3). The minor axis was assumed to vary periodically between the minimum and maximum values, ½(*H*_*β*_)_min_ and ½(*H*_*β,γ*_)_max_, respectively. The subscript *γ* = B indicates the burst mode of drinking, and *γ* = C indicates the continuous mode. For drinking in the continuous mode, we assumed

6a $${a}_{\beta ,{\rm{C}}}(t)=\tfrac{1}{2}{({H}_{\beta })}_{{\rm{\min }}}+\tfrac{1}{4}[{({H}_{\beta ,{\rm{C}}})}_{{\rm{\max }}}-{({H}_{\beta })}_{{\rm{\min }}}]\{1-\,\cos [{\omega }_{{\rm{C}}}(t-{\tau }_{\beta ,{\rm{C}}})]\},$$

where ${\omega }_{{\rm{C}}}=2\pi /{T}_{{\rm{C}}}$ is the average frequency of the pump oscillations with a time period of *T*_C_ and *τ*_*β*,C_ is the time delay of pump *β* in the continuous mode. For the burst mode, we assumed periodic repetition of isolated events. Pump motion in the burst mode was modeled by taking

6b $${a}_{\beta }(t)=\{\begin{array}{c}\tfrac{1}{2}{({H}_{\beta })}_{{\rm{\min }}}\\ \tfrac{1}{2}{({H}_{\beta })}_{{\rm{\min }}}+\tfrac{1}{4}[{({H}_{\beta ,{\rm{B}}})}_{{\rm{\max }}}-{({H}_{\beta })}_{{\rm{\min }}}]\{1-\,\cos [{\omega }_{{\rm{B}},\beta }(t-{T}_{S,\beta })]\}\\ \tfrac{1}{2}{({H}_{\beta })}_{{\rm{\min }}}\end{array}\begin{array}{c}{\rm{for}}\,0\le t\le {T}_{S,\beta },\\ \,\,\,{\rm{for}}\,{T}_{S,\beta }\le t\le {T}_{E,\beta },\\ \,{\rm{for}}\,{T}_{E,\beta }\le t\le {T}_{{\rm{B}}},\end{array}$$

where *T*_*S*_,_*β*_ and *T*_*E*_,_*β*_ are the start and end times, respectively, of the pump operation, ${\omega }_{{\rm{B}},\beta }=2\pi /({T}_{E,\beta }-{T}_{S,\beta })$, is the frequency of the motion for pump *β*, and *T*_*β*_ is the time duration of the burst mode event.

The impedance values are based primarily on the assumption that the flow in the tubes is laminar, steady, and fully developed. The laminar and steady assumptions were rationalized previously. Laminar flow entering a circular tube with a diameter *D* has an entrance length^21,24^ (or development length), *l*_*e*_ ≈ 0.6(Re)*D*, over which the impedance is somewhat larger than for the developed flow in the remainder of the tube. With Re ≈ 0.1 and *D* ≤ 50 μm in the model system, *l*_*e*_ ≤ 3 μm, and flow development effects can be neglected. For straight circular tubes with length *L* and constant diameter *D*, the impedance for steady, laminar, fully developed flow is given by

7a $$\kappa (L,D)=\frac{128\,\mu L}{\pi {D}^{4}}.$$

We assumed that the flow resistance in the feeding canal is independent of flow direction, so that

7b $${K}_{1}(t)=\kappa ({L}_{1},{D}_{1})={\rm{c}}{\rm{o}}{\rm{n}}{\rm{s}}{\rm{t}}{\rm{a}}{\rm{n}}{\rm{t}}.$$

In the pharynx, forward flow is assumed to be through a straight tube with length *L*_2_ and constant diameter *D*_2_. Based on our assumptions about the form and function of the pharyngeal valve, during reverse flow a section of the pharynx with length *l*_2_ constricts to a diameter *d*_2_. We assumed that the pharyngeal valve responds instantaneously to the occurrence of backflow. The time-dependent behavior of the flow impedance was modeled as

7c $${K}_{2}(t)=\{\begin{array}{cc}\kappa ({L}_{2},{D}_{2}) & {\rm{f}}{\rm{o}}{\rm{r}}\,\,{Q}_{2}(t)\ge 0,\\ \kappa ({L}_{2}-{l}_{2},{D}_{2})+\kappa ({l}_{2},{d}_{2}) & {\rm{f}}{\rm{o}}{\rm{r}}\,\,{Q}_{2}(t) < 0.\end{array}$$

In the esophagus, the assumption of no backflow was modeled simply by taking ${K}_{3}\to \infty$ when *Q*_3_ < 0; in practice we used

7d $${K}_{3}(t)=\{\begin{array}{cc}\kappa ({L}_{3},{D}_{3}) & {\rm{f}}{\rm{o}}{\rm{r}}\,\,{Q}_{3}(t)\ge 0,\\ {10}^{6}\,\,\kappa ({L}_{3},{D}_{3}) & {\rm{f}}{\rm{o}}{\rm{r}}\,\,{Q}_{3}(t) < 0,\end{array}$$

which assumes an instantaneous response to backflow.

### Mathematical modeling of power requirements

The mathematical model enabled us to estimate the mechanical power that the feeding system must transfer to the feeding solution in order to produce the pressures and flow rates given by equations (3). This estimate provides a lower bound on the energy per unit time that the mosquito must actually expend during drinking; our model does not account for energy losses in the pumps or for metabolic losses in the muscles and surrounding tissue. At each location on a pump wall, the instantaneous power delivered to the fluid at that location is the product of the normal force applied to the wall, *dF*_*n*_, and the normal velocity of the wall, *v*_*n*_. Again using a quasi-static assumption, the normal force delivered by muscles at a wall location with area *dA* is balanced by the pressure difference across the wall, *dF*_*n*_ = (*p*_hemo_ − *p*_*β*_)*dA*, where *dF*_*n*_(*t*) is defined as positive when pulling to expand the pump. Thus, locally the power was estimated to be

8 $$d{P}_{\beta }(t)={v}_{n}(t)\,d{F}_{n}(t)=-({p}_{\beta }-{p}_{{\rm{hemo}}}){v}_{n}\,dA.$$

The net power supplied by each pump is then given by integrating over the surface area of the pump wall; because we are assuming the pressure in each pump is independent of location, this integral gives

9 $${P}_{\beta }(t)=\int d{P}_{\beta }(t)=-[{p}_{\beta }(t)-{p}_{{\rm{hemo}}}]\int {v}_{n}(t)\,dA=-[{p}_{\beta }(t)-{p}_{{\rm{hemo}}}]\,\,{\dot{V}}_{\beta }(t),$$

where we are again assuming that *p*_hemo_ = *p*_atm_. The total (instantaneous) power delivered to the fluid is then *P*_CP_(*t*) + *P*_PP_(*t*).

Alternatively, the mechanical power required by the fluid to overcome viscous dissipation in the food canal, pharynx, and esophagus can be written as^3^ *P*_*i*_(*t*) = *Q*_*i*_(*t*) Δ*p*_*i*_(*t*). Applying equation (1) and the definitions of Δ*p*_*i*_, and again assuming *p*_food_ = *p*_gut_ = *p*_atm_, gives

10 $${P}_{1}(t)+{P}_{2}(t)+{P}_{3}(t)=-[{p}_{{\rm{CP}}}(t)-{p}_{{\rm{atm}}}]\,\,{\dot{V}}_{{\rm{CP}}}(t)-[{p}_{{\rm{PP}}}(t)-{p}_{{\rm{atm}}}]\,\,{\dot{V}}_{{\rm{PP}}}(t),$$

which yields the same total power as given by *P*_CP_(*t*) + *P*_PP_(*t*) with equation (9).

A negative value of *P*_*β*_(*t*) corresponds to situations in which the pressure in a pump is assisting the motion of the pump wall. There is no physical mechanism whereby this power output from the fluid can be absorbed back into the muscles. Thus, our estimate of the lower bound on the energy per unit time expended by the mosquito should include only *P*_*β*_(*t*) ≥ 0; that is, the input power from the muscles to the fluid in each pump was taken as

11a $${P}_{\beta ,{\rm{in}}}(t)=\,\max \{0,{P}_{\beta }(t)\}.$$

The average energy per unit time required to drive the feeding solution in each mode was then estimated as

11b $${P}_{\gamma }={T}_{\gamma }^{-1}{\int }_{0}^{{T}_{\gamma }}[{P}_{{\rm{CP}},{\rm{in}}}(t)+{P}_{{\rm{PP}},{\rm{in}}}(t)],$$

where *γ* = B denotes the burst mode, *γ* = C denotes the continuous mode, and *T*_*γ*_ is the time period for mode *γ*. Note that for the results reported here, including values of *P*_*β*_(*t*) < 0 when determining *P*_*γ*_ changes the results by less than 3%.

### Mathematical modeling of single-pump feeding

The mathematical model provided a tool for conducting virtual experiments that highlight the effects of parameter variation on system behavior. In particular, we tested system performance when only one of the two pumps was operational. We “knocked-out” one of the two pumps by replacing it with a straight circular tube of length *L*_*β*_ and diameter (*H*_*β*_)_min_. Consider first the case when the pharyngeal pump is removed. The pressure drop between the cibarial pump and the gut, Δ*p*_4_(*t*) = *p*_CP_(*t*) − *p*_gut_, was assumed to be related to the flow rate *Q*_4_(*t*) through the pharynx, pharyngeal pump knockout, and esophagus by equation (2), with ${K}_{4}(t)={K}_{2}(t)+{K}_{{\rm{PP}}}(t)+{K}_{3}(t)$, where *K*_PP_(*t*) is the flow impedance of the pharyngeal pump knockout given by

12 $${K}_{{\rm{PP}}}(t)=\kappa ({L}_{{\rm{PP}}},\,{({H}_{{\rm{PP}}})}_{{\rm{\min }}})={\rm{constant}}.$$

By mass conservation, *Q*_2_(*t*) = *Q*_3_(*t*) = *Q*_4_(*t*) and

13 $${\dot{V}}_{{\rm{CP}}}(t)={Q}_{1}(t)-{Q}_{4}(t).$$

Solving the system of equations (2) and (13) for the case in which the pharyngeal pump is removed gives

14 $$(\begin{array}{c}{Q}_{1}(t)\\ {Q}_{4}(t)\\ {p}_{{\rm{CP}}}(t)\end{array})=(\begin{array}{c}0\\ 0\\ {p}_{{\rm{food}}}\end{array})+\frac{{\rm{\Delta }}{p}_{g}}{\sum {K}_{i}(t)}(\begin{array}{c}-1\\ -1\\ {K}_{1}(t)\end{array})+\frac{{\dot{V}}_{{\rm{CP}}}(t)}{\sum {K}_{i}(t)}(\begin{array}{c}{K}_{4}(t)\\ -{K}_{1}(t)\\ -{K}_{1}(t)\,{K}_{4}(t)\end{array}),$$

where $\sum {K}_{i}(t)={K}_{1}(t)+{K}_{4}(t)$. For the case in which the cibarial pump was removed, the pressure drop between the food source and the pharyngeal pump, Δ*p*_5_(*t*) = *p*_food_ − *p*_PP_(*t*), was assumed to be related to the flow rate *Q*_5_(*t*) through the feeding canal, cibarial pump knockout, and pharynx by equation (2), with *K*_5_(*t*) = *K*_1_(*t*) + *K*_CP_(*t*) + *K*_2_(*t*). The flow impedance of the cibarial pump knockout is given by

15 $${K}_{{\rm{CP}}}(t)=\kappa ({L}_{{\rm{CP}}},\,{({H}_{{\rm{CP}}})}_{{\rm{\min }}})={\rm{constant}}.$$

By mass conservation, *Q*_1_(*t*) = *Q*_2_(*t*) = *Q*_5_(*t*) and

16 $${\dot{V}}_{{\rm{PP}}}(t)={Q}_{5}(t)-{Q}_{3}(t).$$

Solving the system of equations (2) and (16) for the case in which the cibarial pump is removed gives

17 $$(\begin{array}{c}{Q}_{5}(t)\\ {Q}_{3}(t)\\ {p}_{{\rm{PP}}}(t)\end{array})=(\begin{array}{c}0\\ 0\\ {p}_{{\rm{food}}}\end{array})+\frac{{\rm{\Delta }}{p}_{g}}{\sum {K}_{i}(t)}(\begin{array}{c}-1\\ -1\\ {K}_{5}(t)\end{array})+\frac{{\dot{V}}_{{\rm{PP}}}(t)}{\sum {K}_{i}(t)}(\begin{array}{c}{K}_{3}(t)\\ -{K}_{5}(t)\\ -{K}_{3}(t)\,{K}_{5}(t)\end{array}),$$

where in this case $\sum {K}_{i}(t)={K}_{5}(t)+{K}_{3}(t)$. When the cibarial pump is removed, the maximum pressure drop across the proboscis is given by

18 $${\rm{\Delta }}{p}_{\gamma }=\,\max \{|{p}_{{\rm{PP}}}(t)-{p}_{{\rm{food}}}|\}.$$

As before, the details of the knockout systems were determined by specifying the time-dependent variation in the pump volumes and the tube impedance values based on mosquito anatomy and the experimental observations of feeding kinematics in the two-pump system.

## Results and Discussion

### Kinematics of pumping

**Figure 3:**
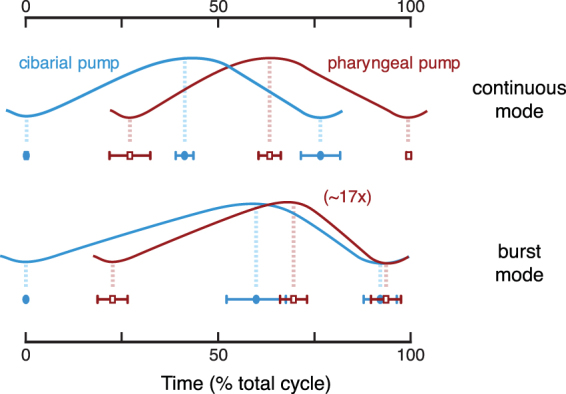
Summary of timing of start, peak, and end of stroke cycles by the cibarial and pharyngeal pumps during continuous and burst mode drinking. Cycle duration is defined from the start of the first pump expansion to the end of the second pump ejection. The data represent the average durations across specimens (Table 1), with error bars representing 95% confidence intervals. The plots represent the volume filling of each anatomical pump through the cycle; for burst mode, the pharyngeal pump volume expansion is approximately 17 times greater than the cibarial pump expansion.

Analysis of the image intensity values from x-ray videos enabled us to determine the timing of filling and ejection of pumps within a cycle, revealing two distinct modes of drinking (Figs 1e,f, 3). The dominant mode was continuous pumping (Fig. 1e and Supplemental Videos S1, S2), in which the two pumps reciprocated cyclically with similar stroke dynamics, with fill-ejection cycles offset by 27 ± 6% (91 ± 40 ms, mean ± S.D.; 6 animals, 126 cycles; p < 0.0001; Fig. 3). In this mode, we observed that the cibarial pump begins the cycle with expansion, followed by expansion of the pharyngeal pump. The cibarial pump then ejects its contents while the pharyngeal pump is still expanding. As the pharyngeal pump contracts and ejects fluid into the esophagus, completing the cycle, the cibarial pump begins its next round of expansion. This description of continuous feeding is consistent with previous descriptions of pumping in mosquitoes, including pumping frequencies (4.0 ± 1.0 Hz, Table 1) that overlap those from studies using x-rays and other techniques (~2–12 Hz^9,12,13,27,28^). Based on its occurrence here and in previous studies, continuous pumping appears to be the dominant mode of drinking in mosquitoes.

**Figure 4:**
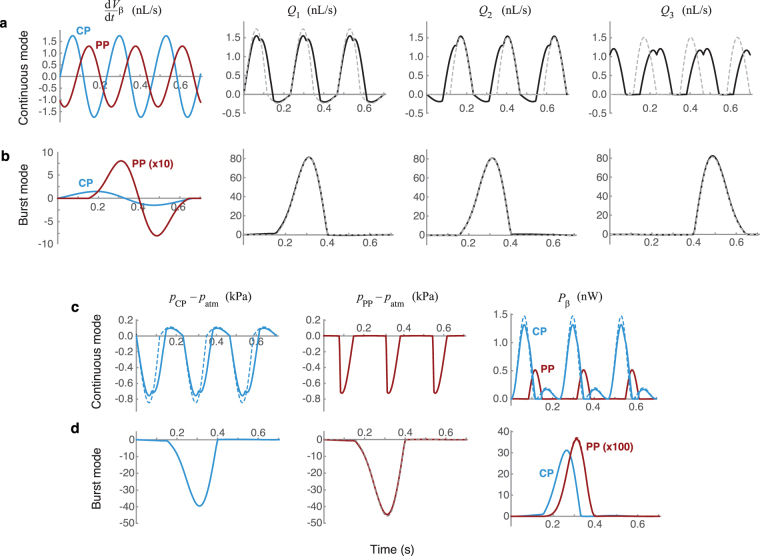
Results from fluid mechanical modeling of the feeding system. (**a,b**) Rates of volume change, ${\dot{V}}_{\beta }$, and flow rates, *Q*_*i*_, in the model system (in nanoliters per second) are shown as a function of elapsed time (in seconds). Rows are labeled according to the pumping mode, and columns are labeled according to the dependent variable being plotted. Subscripts 1–3 refer to the feeding canal, pharynx, and esophagus, respectively, corresponding to lengths labeled *L*_1_ − *L*_3_ in Fig. 2c. Single-pump model results are shown with dashed lines in the panels for *Q*_𝛼_. For the continuous mode, the single pump is the cibarial pump, and in the burst mode the single pump is the pharyngeal pump. In the burst mode cases, the single-pump results are nearly indistinguishable from the two-pump results. (**c,d**) Pressure variation within the two pumps relative to atmospheric pressure (*p*_*β*_ − *p*_atm_, in kPa) and the instantaneous power added to the fluid (*P*_*β*_, in nanowatts) as functions of time. Rows are labeled according to the pumping mode, and columns are labeled according to the dependent variable being plotted. Single-pump model results are shown with dashed lines; in the burst mode cases, the single-pump results are nearly indistinguishable from the two-pump results.

Morphologically, the pharyngeal pump is larger than the cibarial pump, and its potential expandable capacity is also larger, as suggested previously from a single sacrificed specimen using µCT imaging^9^. However, our x-ray data indicate that the pharyngeal pump expands by only a small amount during continuous pumping (Fig. 1c and Supplemental Videos S1, S2), with expansion confirmed by the displacement of adjacent tracheal tubes. The volume change in the lumen appears to be similar for both pumps, suggesting that the main function of the pharyngeal pump is to pass fluid food from the cibarial pump and push it into the esophagus. This hypothesis is supported by the knock-out modeling, which reveals very little change in performance when the pharyngeal pump is removed from the system (Fig. 4).

We also newly observed a second mode of pumping, in which the pumps expand to a much greater extent (Fig. 1d). In the x-ray videos, this mode of pumping appeared as a burst of activity, with an event consisting of a single fill/ejection stroke of each pump (Fig. 1f and Supplemental Videos S3, S4). Similar to continuous pumping, a cycle in the ‘burst’ mode begins by the expansion of the cibarial pump, with the expansion of the pharyngeal pump following 23 ± 7% of a cycle later (152 ± 67 ms; 10 animals, 31 cycles; p < 0.0001; Fig. 3). However, burst mode pumping was characterized by three features that differ substantially from continuous pumping. First, the volume expansion was much greater for both pumps, with an estimated increase in the maximum pump volume by a factor of 1.6 (cibarial pump) and 19 (pharyngeal pump) relative to continuous pumping (Table 4). Second, the maximum expansion of each pump occurred closer in time than in continuous pumping (offset between pumps, 49 ± 61 ms vs. 69 ± 24 ms; p = 0.04), and the completion of ejection occurred simultaneously (difference between end times, 16 ± 98 ms, p = 0.34). Lastly, the total cycle duration was more than twice as long as for continuous pumping (703 ± 288 vs. 319 ± 91 ms; p < 0.0001). These features resulted in a much greater average volume flow rate for the burst mode (16 vs. 0.58 nL/s, based on modeling results; see below).

**Table 4:** Estimated minimum and maximum volumes of the head pumps during continuous and burst mode pumping. These values were calculated in the mathematical model (Eq. 4) using morphological dimensions from Table 3.

	Continuous mode pumping		Burst mode pumping	
	CP	PP	CP	PP
Minimum volume (nL)	0.18	0.33	0.18	0.33
Maximum volume (nL)	0.30	0.43	0.48	7.96
Max volume change (nL)	0.12	0.10	0.30	7.63
Volume change relative to continuous mode	1	1	2.5	76.3

There was no clear pattern to the occurrence of bursts. Most burst events (n = 20 of 31) occurred within bouts of continuous pumping (e.g., Fig. 1f), but some occurred at the start (n = 3) or end (n = 5) of a bout, or both (n = 1). Isolated bursts with no associated continuous pumping were also observed (n = 2). Because the burst mode produces a much higher instantaneous flow rate than does continuous pumping, a mosquito using only the burst mode could theoretically complete a drinking bout in a much shorter time, which should be ecologically advantageous for avoiding detection or predation. However, the intermittent usage of this mode led us to question whether mechanical tradeoffs exist that reduce its effectiveness as a mechanism for normal drinking. Specifically, we asked if the usage rates of the two observed behaviors could be rationalized by the physics of flow through the system.

### Modeling of pumping

The quantities needed to discuss the flow physics, namely the flow rates and internal pressures, are difficult or impossible to measure directly with current non-invasive measurement techniques. To address this issue, we developed a mathematical model of drinking a Newtonian fluid at atmospheric pressure, based on the dynamics of pumping observed in the live mosquitoes (drinking a sugar-water solution, as in our experiments). Solving the model’s equations provided time-varying values for the pressures and flow rates in the system, and enabled calculation of the power required to drive the flow.

Time-dependent modeling results are shown in Fig. 4. Pressure boundary conditions for these calculations were assumed to be *p*_food_ = *p*_gut_ = *p*_atm_. The rates of volume change in the pumps, ${\dot{V}}_{\beta }(t)$, were determined by the fit to the experimental data and time derivatives of equations (5) and (6). For the continuous mode, the cycle period was taken to be the average of the two pump durations in Table 1, giving *T*_C_ = 233.5 ms. The model cycle was assumed to coincide with the start of the cibarial pump cycle, so that τ_CP,C_ = 0 ms. The time delay for the pharyngeal pump was assumed to be the average of the differences between the pump start and end times, giving τ_PP,C_ = 83.5 ms. For the burst mode, the measurements from Table 1 were used to determine *T*_B_ = 703 ms, *T*_S,CP_ = 0.4 ms, *T*_E,CP_ = 662 ms, *T*_S,PP_ = 153 ms, and *T*_E,PP_ = 646 ms. Instantaneous flow rates through the food canal, pharynx, and esophagus and instantaneous pressures in the cibarial and pharyngeal pumps were determined by solving equations (3). Although the instantaneous flow rates vary through the system, by mass conservation the time-averaged flow rates for a given pumping mode are identical in each part of the system when averaged over a complete drinking cycle.

Note that drinking in the continuous mode results in periodic backflow through the food canal and out of the mosquito. The maximum backflow rate, corresponding to negative values of *Q*_1_, was found to be approximately 13% of the maximum inflow rate. Similarly, the volume of backflow out of the mosquito’s food canal was calculated to be 9% of the net inflow volume. This level of backflow is consistent with the experimental observations shown in Fig. 4 of Kim *et al*.^12^.

The mechanical performance of the continuous and burst modes can be compared by considering the time-averaged flow rate, *Q*_*γ*_, the maximum pressure drop across the proboscis, Δ*p*_*γ*_, and the power added to the fluid by the pumps, *P*_*γ*_. For the continuous mode, the average flow rate in the model is *Q*_C_ = 0.58 nL/s, the maximum pressure drop across the proboscis is Δ*p*_C_ = 0.76 kPa, and the two pumps combine to add *P*_C_ = 0.48 nW of power to the fluid. For the burst mode, these values are much higher—the average flow rate in the model is *Q*_B_ = 16 nL/s, the maximum pressure drop across the proboscis is Δ*p*_B_ = 40 kPa, and the pumps add *P*_B_ = 530 nW of power to the fluid.

The nonlinear relationship between pressure and flow rate in this low Reynolds number fluid system is due to the time dependence of the driving kinematics. By way of illustration, consider flow through a single straight circular pipe with diameter of 25 μm and length of 6.4 mm. A constant pressure drop of 1 kPa will produce a steady flow rate of 0.5 nL/s. If instead the pressure drop across the pipe changes periodically, with a value of 40 kPa for 0.8 seconds followed by a value of 0 kPa for 0.2 seconds, the average flow rate will be 16 nL/s.

Thus, while the burst mode provides a ~27× increase in volume flow rate, the nonlinear relationship between pressure and flow rate means that this boost comes at a substantial cost. The high flow rate requires a pressure drop that is ~53× greater than in continuous pumping, resulting in a massive ~1,100× increase in power expenditure. Overall, the effectiveness of burst mode pumping, calculated as the ratio of the average flow rate to the power expended in a single cycle, is over 40× lower than in continuous mode pumping. If power were the only consideration, it would be advantageous for the mosquito to solely use continuous pumping.

### Virtual knock-outs of individual pumps

These results led us to further probe the relative roles of the two pumps during drinking. We removed one pump or the other in the mathematical model, a knock-out procedure that served to isolate the effects of each pump on ingestion performance. Of the four possible knock-out scenarios, we focused on the following two representative cases: (1) continuous mode drinking using only the cibarial pump, and (2) burst mode drinking using only the pharyngeal pump. In both cases, operating with only one pump showed very little change relative to two-pump operation (Fig. 4). In the continuous mode, the timing of the flow into the gut is changed slightly by relying solely on the cibarial pump. Despite the absence of the larger pharyngeal pump, the peak flow rate through the food canal is increased by 12% and the average volume flow rate is reduced by 18%, with a corresponding reduction in overall power consumption by 14%. In the burst mode, removal of the cibarial pump produced an even weaker effect—its removal reduced the average flow rate by less than 2% while increasing power expenditure by less than 1%. These results suggest that the pharyngeal pump plays only a minor role in continuous drinking, and the importance of the cibarial pump is almost negligible for burst mode drinking. In each of the two modes, a different pump dominates the fluid mechanics of the system.

### Behavioral role of the burst mode

Although the behavioral role of the burst mode is not known, the experimental and modeling results provide the basis for new hypotheses relating form to function. The massive expansion of the pump lumen during a burst event suggests that the muscles driving pump expansion are recruited fully only during this mode. This expansion creates a very large pressure drop across the proboscis, which might be used to clear a blockage in the system (the “clearance” hypothesis) or to prime the food canal at the start of feeding (the “priming” hypothesis). To our knowledge, neither hypothesis has received concerted study. However, blockages by bacteria and other microbial cells have been observed to occur in fluid-feeding sharpshooters, which may cause the precibarial valve to “stick” open, necessitating egestion behaviors to clear the blockage^29^.

**Figure 5:**
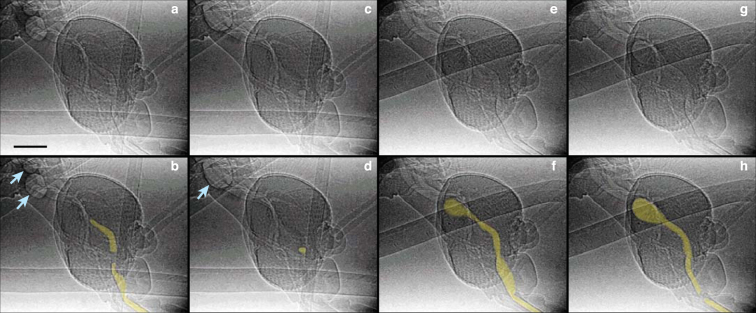
Presence of air in the feeding system associated with burst mode pumping. Images are cropped from video frames of Supplementary Video S6. Top and bottom image pairs are the same image, with the location of air emphasized in the bottom row—yellow coloring highlights air in the feeding system, and blue arrows point to bubbles in the foregut. The other air-filled structures are tracheal tubes, which can also be seen (in green) in Supplementary Video S5. (**a,b**) Air in the proboscis, cibarial pump, and pharynx just after a burst mode event. Note the gap between the cibarial pump and pharynx, which most likely represents the location of the pharyngeal valve in its closed position. (**c,d**) A small amount of air remains in the anterior pharynx, 4.3 s after the previous frame. The large bubble in the foregut (blue arrow) is composed of air that had just been pumped through the feeding system. Estimates of the volume of air in such bubbles are consistent with volume calculations from the pumps, confirming that the model’s shape assumptions in Equation (5) are reasonable. (**e,f**) Air throughout the feeding system during a different burst mode pumping event. At this point in the cycle, the cibarial pump is at its maximum expanded state, while the pharyngeal pump is still expanding. (**g,h**) Air in the system during the same burst mode event, 67 ms after the previous image. Here the pharyngeal pump is in its maximum expanded state within this cycle, and the cibarial pump is contracted. Note that for (**e–h**), bubble size does not necessarily represent the maximum volume of the system, because liquid food may still be present. The meniscus in the food canal in (**h**) suggests that the food canal surface is hydrophilic. Scale bar, 200 µm.

Although little is known about blockages in fluid-feeding insects, the presence of air in the system may provide a major mechanical challenge. In our recordings, we observed multiple instances of air in the feeding system, which the mosquito overcame by pumping (Supplementary Video S6), thereby passing the air to the gut (Fig. 5). Six sequences from two specimens exhibited transport of air associated with burst events. In five sequences, transport of the air was preceded by a burst event, and in one sequence, transport of air occurred just prior to the burst event. These results suggest that the burst mode might be used to clear air from the upstream portion of the feeding system. However, the observation of air ingestion during continuous drinking prior to a burst event demonstrates that this mode of pumping may not be strictly required for every blockage condition. The location of the air in system and the volume of air encountered are likely to be important variables in this regard, but our setup with a single x-ray view did not allow us to simultaneously image both the pumps in the head and the status of the food canal throughout the proboscis, a topic for future study.

To explore the potential value of the burst mode for clearance of air, we considered the effect of encountering air at the proboscis inlet during drinking. Based on observed properties of the butterfly proboscis^30,31^, the function of the mosquito proboscis, and evidence from our video analysis (see Fig. 5 and Supplementary Video S6), we assumed that the feeding solution wets the surface of the food canal, so that capillarity aids in priming the food canal at the start of drinking. However, if the food canal is full of feeding solution and is then exposed to air (such as a large bubble, or at the completion of drinking), a meniscus forms that resists being pulled up the food canal. The pressure drop across the meniscus surface was taken to be^21^ Δ*p*_*m*_ = 2*σ*/*R*, where *R* is the radius of curvature for the surface and *σ* = 0.077 N/m is the assumed surface tension of the feeding solution in air^32,33^. Assuming the air at the end of the proboscis is at atmospheric pressure, the feeding solution at the tip of the feeding canal has a pressure *p*_inlet_ = *p*_atm_ − Δ*p*_*m*_, which is lower than atmospheric pressure and the hemolymph pressure. In order to draw the meniscus toward its head, the mosquito must overcome the pressure difference caused by the meniscus *and* the viscous resistance in the food canal, requiring *p*_CP_ < *p*_inlet_. Assuming *R* = *D*_1_/2 to be the same as the radius of the food canal gives *p*_CP_ − *p*_atm_ < −Δ*p*_*m*_ = −6.2 kPa. As shown in Fig. 4, our model results demonstrate that a pressure drop of this magnitude across the proboscis is generated only by the burst mode and cannot be generated by the continuous mode. Given these assumptions, the burst mode must be used to draw the remaining food up the food canal.

Alternatively, the large bursts of pumping could be used to provide quick boosts in volume flow rate, enabling faster drinking bouts that would help the mosquito to avoid detection. Mosquitoes infected with malaria take larger blood meals^34^, suggesting that mechanisms that enable faster drinking may affect transmission of mosquito-borne diseases including West Nile, yellow fever, dengue, Chikungunya, and Zika, which impact hundreds of thousands of people each year^35,36^. However, it is not known if mosquitoes employ the burst mode when drinking blood. Blood is non-Newtonian, whose differing properties may influence the kinematics of pumping by female mosquitoes. Furthermore, in contrast to drinking from a still feeding solution at atmospheric pressure, the pressure of a host’s blood (~1.3–5.3 kPa in the capillaries of a human host^37^, for example) may assist the mosquito in drinking. Such behavioral questions and their links to ecology remain to be addressed.

## Conclusions

The previous understanding of feeding by mosquitoes assumed that both pumps in the head work at full capacity during feeding, but the discovery of the burst mode demonstrates that stroke volume during continuous drinking is only partially utilized—leaving a large capacity in reserve—and that pump timing can be modulated dramatically across cycles. The virtual knockout results suggest that a single pump can accomplish the desired system performance in each mode of drinking—the cibarial pump in continuous mode pumping, and the pharyngeal pump in burst mode pumping. Thus, the cibarial and pharyngeal pumps appear to switch mechanical roles between the two modes of pumping. This ability to switch roles affords the mosquito a dual-function system, one that is able to ingest efficiently during continuous pumping, but also able to create a temporary boost in performance using the burst mode. Furthermore, the existence of two pumps allows one or the other to dominate the flow production, representing a new mechanical rationale for a two-pump system in insects.

Overall, our results on mosquitoes suggest that the advantage of a two-pump system may lie in its flexibility of mechanical function—the dynamics of pumping can be modified to substantially alter feeding performance, an ability that may be utilized in other insects with a two-pump system, including flies, ants, and bees^38^. Some other fluid-feeding insects, such as lepidopterans and hemipterans, possess only one large pump in the head^23,39^, but such systems include other components, including upstream and downstream valves and additional muscles encompassing the pump^40^. These morphological features may compensate for the lack of a second pump and perhaps provide the additional functionality afforded by a two-pump system.

## Electronic supplementary material

Supplementary Material

Video S1

Video S2

Video S3

Video S4

Video S5

Video S6
