# Supplementary material for: Burst mode pumping: A new mechanism of drinking in mosquitoes
Source: Sci Rep. 2018 Mar 20;8:4885. doi: 10.1038/s41598-018-22866-w (PMC5861067; doi:10.1038/s41598-018-22866-w)
Supplement: Supplementary file 1 — Supplementary Material [file 41598_2018_22866_MOESM1_ESM.pdf]

Supplementary Materials for:

Burst mode pumping: A new mechanism of drinking in mosquitoes

Kenji Kikuchi, Mark A. Stremler, Souvick Chatterjee, Wah-Keat Lee, Osamu Mochizuki, John J. Socha*

*Correspondence to: Jake Socha, jjsocha@vt.edu

Contents:

Figures S1, S2
Video legends S1 to S6
Notation

Supplementary figures

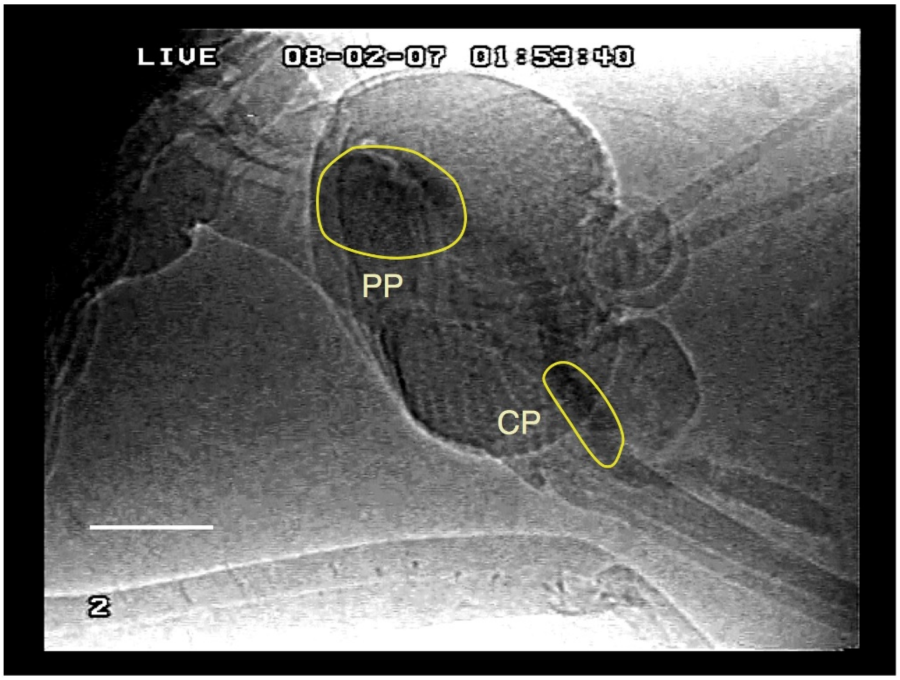

Figure S1. Representative locations of the regions of interest (ROI) for the cibarial (CP) and pharyngeal (PP) pumps. Scale bar, 200 μm.

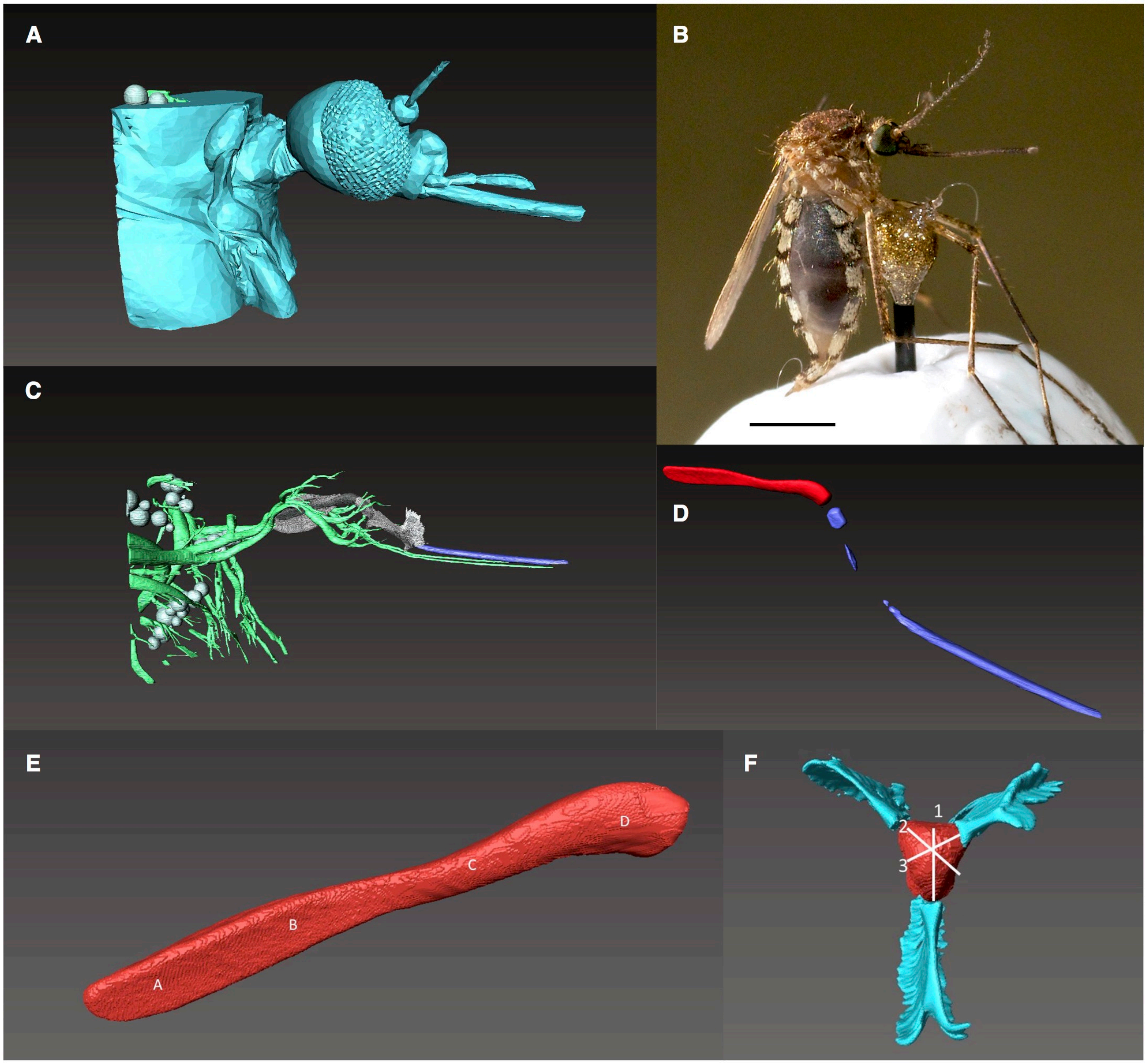

Figure S2. Tomography was used to obtain dimensions of the internal lumen of the pump system. Original scans were conducted at beamline 2-BM of the Advanced Photon Source, Argonne National Laboratory. (**a**) Three-dimensional rendering of the anterior thorax, head, and partial proboscis of an *Aedes vexans* sample, shown in mounted position for scanning in (**b**). Also see movie S5. (**c**) Tracheal tubes (green), cuticular structures surrounding the cibarial and pharyngeal pumps (grey), and food canal (purple). The white spheres are bubbles in the gut. (**d**) Food canal (purple) and pharyngeal pump (red). (**e**) Four locations (A-D) showing locations of measurement to obtain lumen width. Measurements were taken and averaged at three cross-sections (1-3) shown in (**f**). The light blue represents the internal cuticle attached to the pump. Scale bar in (B), 1 mm.

Video legends

Video S1. Representative video of pumping in the head of the mosquito *Aedes vexans* during a bout of continuous drinking. The changes in intensity in the food transport system represent the movement of sugar water that has been marked with iodine to facilitate visualization. Note the congruent movement of tracheal tubes adjacent to the pharyngeal pump as it changes volume cyclically. The field of view is 1.3 x 1.0 mm (2.24 μm/px). Video (30 Hz) recorded at beamline 32-ID at Argonne National Laboratory using synchrotron x-rays.

Video S2. All sequences of continuous mode pumping analyzed in this study. The field of view is 1.3 x 1.0 mm (2.24 μm/px). Video (30 Hz) recorded at Argonne National Laboratory using synchrotron x-rays.

Video S3. Representative video of burst mode pumping in the head of the mosquito *Aedes vexans*. Note the massive increase in volume in the pharyngeal pump during the burst event. The field of view is 1.3 x 1.0 mm (2.24 μm/px). Video (30 Hz) recorded at Argonne National Laboratory using synchrotron x-rays.

Video S4. All burst mode events analyzed in this study. The field of view is 1.3 x 1.0 mm (2.24 μm/px). Video (30 Hz) recorded at Argonne National Laboratory using synchrotron x-rays.

Video S5. Three-dimensional rendering of a mosquito revealing the pumping apparatus in the head of an *Aedes vexans* mosquito. The green tubes are tracheal tubes, the white spheres are bubbles in the gut, the gray structures surrounding the pumps are internal cuticles, and the purple cylinder is the feeding canal leading to the cibarial and pharyngeal pumps. Tomography conducted at beamline 2-BM at Argonne National Laboratory.

Video S6. Burst mode pumping events associated with air in the feeding system. Six sequences are shown from two *Aedes* mosquitoes (three from each specimen). In sequences 1 to 5, air is present in some part of the system prior to the occurrence of a burst mode pumping event. In sequence 6, the air is first cleared (i.e., passed to the foregut), and then a burst mode pumping

event occurs. The field of view is 1.3 x 1.0 mm (2.24 μm/px). Video (30 Hz) recorded at Argonne National Laboratory using synchrotron x-rays.

Notation

Below we provide a summary of the abbreviations used in the mathematical modeling of pumping in the main text of the paper.

α	Womersley number
β	Subscript indicating the cibarial pump (CP) or pharyngeal pump (PP)
$\Delta p_i(t)$	Time-dependent pressure drop over the length of tube *i*
Δp_m	Pressure difference across a meniscus due to surface tension
γ	Subscript indicating the burst mode (B) or continuous mode (C) of pump operation
μ	Dynamic viscosity of the sugar/iodine solution
ρ	Density of the sugar/iodine solution
σ	Surface tension of the feeding solution
$a_\beta(t)$	Time-dependent semi-minor axis of pump β
b_β	Semi-major axis of pump β
d_2	Diameter of pharyngeal valve region when closed
D_i	Diameter of tube *i*
H_β	Height of pump β transverse to the flow
$(H_\beta)_{\min}$	Minimum height of pump β for both pumping modes
$(H_{\beta,\gamma})_{\max}$	Maximum height of pump β in pumping mode γ

i	Index representing the feeding tube (i=1), pharynx (i=2), or esophagus (i=3)
$K_i(t)$	Time-dependent flow resistance in tube i
K_i	Constant value of $K_i(t)$ used when valves in tube i are open
K_i'	Constant value of $K_i(t)$ used when valves in tube i are closed
l_2	Length of pharyngeal valve region
L_i	Length of tube i
L_β	Length of pump β in direction of flow
p_{atm}	Atmospheric pressure
$p_\beta(t)$	Time-dependent pressure in pump β
$P_\beta(t)$	Time-dependent instantaneous power added to and/or extracted from the fluid by pump β
$P_{\beta,\text{in}}(t)$	Time-dependent instantaneous power delivered into the fluid by pump β
p_{inlet}	Pressure at the inlet tip of the food canal
Q_i	Instantaneous flow rate through tube i
R	Radius of curvature
Re	Reynolds number
T	Characteristic time scale for the flow
T_B	Time duration of the burst mode pumping cycle

T_C	Time period for the continuous mode pumping cycle
T_D	Time delay between cibarial and pharyngeal pump cycles in continuous mode pumping
$T_{E,\beta}$	End time of pump β operation in burst mode
$T_{S,\beta}$	Start time of pump β operation in burst mode
U	Average fluid velocity in the feeding canal
$V_\beta(t)$	Time-dependent volume of the prolate spheroid representing pump β
x_L	Entrance length for developing flow
